# Influenza A Virus Host Shutoff Disables Antiviral Stress-Induced Translation Arrest

**DOI:** 10.1371/journal.ppat.1004217

**Published:** 2014-07-10

**Authors:** Denys A. Khaperskyy, Mohamed M. Emara, Benjamin P. Johnston, Paul Anderson, Todd F. Hatchette, Craig McCormick

**Affiliations:** 1 Department of Microbiology and Immunology, Dalhousie University, Halifax, Nova Scotia, Canada; 2 Laboratory of Stem Cell Research, Qatar Biomedical Research Institute, Doha, Qatar; 3 Department of Virology, School of Veterinary Medicine, Cairo University, Giza, Egypt; 4 Division of Rheumatology, Immunology and Allergy, Brigham and Women's Hospital, Harvard Medical School, Boston, Massachusetts, United States of America; Johns Hopkins University - Bloomberg School of Public Health, United States of America

## Abstract

Influenza A virus (IAV) polymerase complexes function in the nucleus of infected cells, generating mRNAs that bear 5′ caps and poly(A) tails, and which are exported to the cytoplasm and translated by host machinery. Host antiviral defences include mechanisms that detect the stress of virus infection and arrest cap-dependent mRNA translation, which normally results in the formation of cytoplasmic aggregates of translationally stalled mRNA-protein complexes known as stress granules (SGs). It remains unclear how IAV ensures preferential translation of viral gene products while evading stress-induced translation arrest. Here, we demonstrate that at early stages of infection both viral and host mRNAs are sensitive to drug-induced translation arrest and SG formation. By contrast, at later stages of infection, IAV becomes partially resistant to stress-induced translation arrest, thereby maintaining ongoing translation of viral gene products. To this end, the virus deploys multiple proteins that block stress-induced SG formation: 1) non-structural protein 1 (NS1) inactivates the antiviral double-stranded RNA (dsRNA)-activated kinase PKR, thereby preventing eIF2α phosphorylation and SG formation; 2) nucleoprotein (NP) inhibits SG formation without affecting eIF2α phosphorylation; 3) host-shutoff protein polymerase-acidic protein-X (PA-X) strongly inhibits SG formation concomitant with dramatic depletion of cytoplasmic poly(A) RNA and nuclear accumulation of poly(A)-binding protein. Recombinant viruses with disrupted PA-X host shutoff function fail to effectively inhibit stress-induced SG formation. The existence of three distinct mechanisms of IAV-mediated SG blockade reveals the magnitude of the threat of stress-induced translation arrest during viral replication.

## Author Summary

Like all viruses, Influenza A virus (IAV) is absolutely dependent on host-cell protein synthesis machinery. This dependence makes the virus vulnerable to the innate ability of cells to inhibit protein synthesis in response to various types of stress. This inhibition, termed translation arrest, helps cells survive adverse conditions by re-dedicating their energy to stress responses. When cells arrest translation, they form stress granules: depots of untranslated mRNAs and associated proteins. Translation arrest and formation of stress granules can be induced pharmacologically, and in this work we sought to determine whether stress granule induction would be effective in blocking IAV replication. Here we demonstrate that treatment of cells with inducers of stress granules at early times after infection resulted in blockade of viral protein synthesis and stopped viral replication. At later times post-infection, by contrast, IAV proteins prevented pharmacological induction of stress granules. We identified three viral proteins – more than in any virus to date – that work in concert to prevent stress granule formation. Taken together, our studies reveal a multipronged approach for viral suppression of translation arrest, and identify a window of opportunity early in infection when pharmacological induction of stress granules has a strong antiviral effect.

## Introduction

Transcription of Influenza A virus (IAV) genes is performed by a viral polymerase that generates 5′-capped and polyadenylated (poly[A]) messenger RNAs (mRNAs) structurally similar to host mRNAs [1]. Despite this similarity, IAV transcripts gain preferential access to cellular translation machinery through a host shutoff mechanism executed by the viral non-structural protein 1 (NS1) [2], [3] and the recently discovered viral PA-X protein [4].

The reliance on cap-dependent translation initiation makes viral mRNAs susceptible to host-cell stress-induced translation inhibition mechanisms. This inhibition results from phosphorylation of eukaryotic translation initiation factor-2α (eIF2α) by any of four kinases activated by distinct types of stress [5]. Heme-regulated translation inhibitor (HRI) kinase is activated in response to oxidative stress, GCN2 senses nutrient deprivation and ultraviolet damage, double-stranded RNA (dsRNA)-dependent protein kinase R (PKR) is activated in response to viral infections, and the PKR-like endoplasmic reticulum kinase (PERK) signals in response to endoplasmic reticulum stress. Inhibition of translation initiation leads to runoff of elongating ribosomes from mRNA and the accumulation of stalled translation preinitiation complexes. Translationally inactive messenger ribonucleoproteins (mRNPs) recruit RNA-binding proteins with self-aggregating properties, including the T-cell intracellular antigen 1 (TIA-1), TIA-1-related protein (TIAR), and ras GTPase-activating protein-binding protein 1 (G3BP1), which nucleate the formation of large cytoplasmic mRNP foci known as stress granules (SGs; [6]). Many viruses have evolved specific mechanisms that modulate SG responses (reviewed in [7]).

Previously we demonstrated that SGs do not form at any point during IAV infection [8]. Importantly, complete inhibition of SG formation is dependent on NS1. In cells infected with NS1-mutant viruses, SG formation is triggered by PKR activation. However, more than 50% of cells infected with NS1-mutant viruses remained SG-free and allowed IAV replication cycle progression, suggesting the existence of additional NS1-independent mechanisms of SG suppression. In this work, by analyzing SG formation in IAV-infected cells in response to a variety of stresses, we report a robust mechanism of SG inhibition that becomes engaged at later times post-infection and acts despite strong eIF2α phosphorylation. Maximal SG inhibition coincided with a striking depletion of cytoplasmic poly(A) mRNA and the nuclear re-localization of poly(A)-binding protein 1 (PABP1) at later stages of viral replication, effects reminiscent of host shutoff mechanisms observed in other viral systems [9]–[11]. Screening of known IAV ORFs derived from A/PuertoRico/8/34(H1N1) strain (PR8) revealed the identities of two additional viral SG inhibitors, nucleoprotein (NP) and polymerase-acidic protein-X (PA-X), which both act independently of eIF2α phosphorylation. Furthermore, we provide evidence that in the early stages of infection, before the accumulation of sufficient quantities of SG-inhibiting proteins, viral replication is vulnerable to translation-inhibiting drugs that induce SGs and block viral replication.

## Results

### Influenza A virus inhibits SG formation by an NS1-independent mechanism

**Figure 1:**
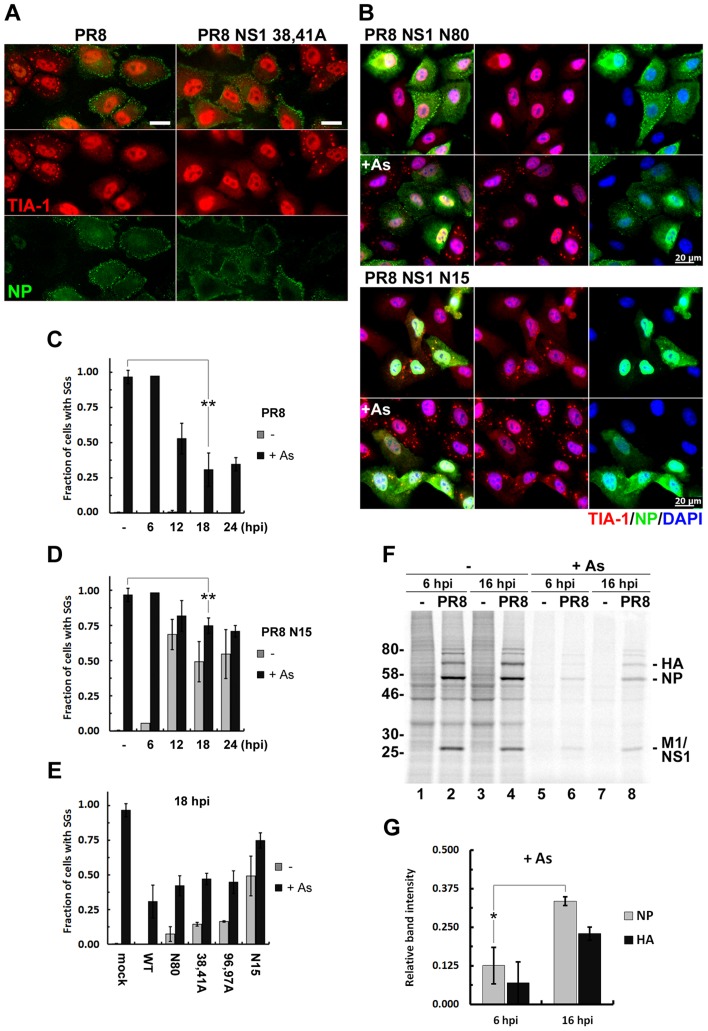
Influenza A virus inhibits sodium arsenite-induced SG formation at later times post-infection by an NS1-independent mechanism. (A and B). SG formation was analysed using immunofluorescent staining of A549 cells infected with the wild type (PR8) and the indicated NS1-mutant influenza A viruses treated with sodium arsenite (As) at 18 hours post-infection (hpi). Virus-infected cells were identified by antibody staining for NP (green) and SGs were visualized using staining for TIA-1 (red). In panel (B) nuclei were stained with DAPI (blue). Scale bars represent 20 µm. (C) Virus and arsenite-induced SG formation was quantified in A549 cells infected with WT PR8 virus at 6, 12, 18 and 24 hours post-infection (hpi). (D) Same quantification as in (C) was done in cells infected with PR8 NS1 N15 virus. (E) SG formation at 18 hpi was compared in mock-infected cells and cells infected with recombinant PR8 viruses with the indicated NS1 mutations. (F) Autoradiogram of the whole cell lysates of mock and PR8 virus-infected A549 cells. At 6 or 16 hpi cells were either left untreated (-) or treated with sodium arsenite (+As) for 1 hour and then immediately pulse-labelled with [^35^S]methionine-[^35^S]cysteine for 30 min. (G) Viral NP and HA translation rates post-arsenite treatment are compared between 6 and 16 hpi. Band intensities after arsenite treatment from (F) were normalized to untreated sample band intensities.

Previously we have observed SG formation triggered by infection with recombinant influenza viruses with amino acid substitutions in NS1 that compromise its ability to counteract PKR [8]. For two different NS1 mutant viruses, SG formation peaked at 18 hpi; however, a majority of infected cells remained SG-free throughout a 24 h time course, suggesting that either these cells are not competent to form SGs, or that IAV has additional mechanisms that block PKR activity and/or SG formation. To test whether these infected cells are competent to form SGs, we treated cells infected with either WT or NS1 mutant viruses with sodium arsenite, a potent inducer of SGs that activates the oxidative stress-responsive eIF2α kinase HRI [12]. By 18 hpi, sodium arsenite had induced the formation of large, well-defined SGs in the cytoplasm of all uninfected cells, while most of the cells infected with either WT or NS1 dsRNA binding mutant viruses failed to form SGs (Fig. 1A). We reinforced these observations through use of an additional NS1-mutant virus whose NS1 protein retains the functional dsRNA-binding domain but completely lacks the C-terminal effector domain (PR8 NS1 N80; Fig. 1B upper panel), and a mutant virus completely lacking functional NS1 (PR8 NS1 N15). The latter virus triggered formation of PKR-mediated SGs, but was still able to partially inhibit arsenite-induced SG formation at 18 hpi (Fig. 1B lower panel, and Fig. 1D).

We observed striking inhibition of arsenite-induced SGs in WT PR8-infected cells from 12 hpi onwards, peaking at 18 hpi (Fig. 1C). For this reason, we evaluated a panel of NS1-mutant viruses at this time point (Fig. 1E). These NS1 mutant viruses, while triggering SG formation to varying degrees in infected cells, all inhibited arsenite-induced SGs compared to mock-infected cells. Furthermore, this effect was not cell-line specific, as we observed the same magnitude of SG inhibition by PR8 in U2OS osteosarcoma cells (Fig. S1A–M). Importantly, SG inhibition did not result from blockade of arsenite-induced eIF-2α phosphorylation (Fig. S1N), and the levels of SG constituent proteins TIA-1, G3BP, PABP1 and eIF4A did not change throughout a 24 h infectious cycle (Fig. S1O).

What is the significance of SG inhibition at later times post-infection? Using pulse-labeling experiments, we queried the rate of viral protein synthesis and sensitivity to sodium arsenite treatment. We observed a high rate of viral protein synthesis, which was maintained at later times post-infection (Fig. 1F lanes 1–4). Sodium arsenite treatment inhibited the production of viral proteins at 6 hpi and 16 hpi; however, the remaining translation rate was significantly higher at 16 hpi (Fig. 1F, G), coincident with SG inhibition. Collectively, our data suggest that at later times post-infection the virus establishes an environment in which accumulation of viral proteins becomes less sensitive to stress-induced translation arrest.

### SG inhibition is independent of vRNP export and coincides with depletion of cytoplasmic poly(A) RNA

**Figure 2:**
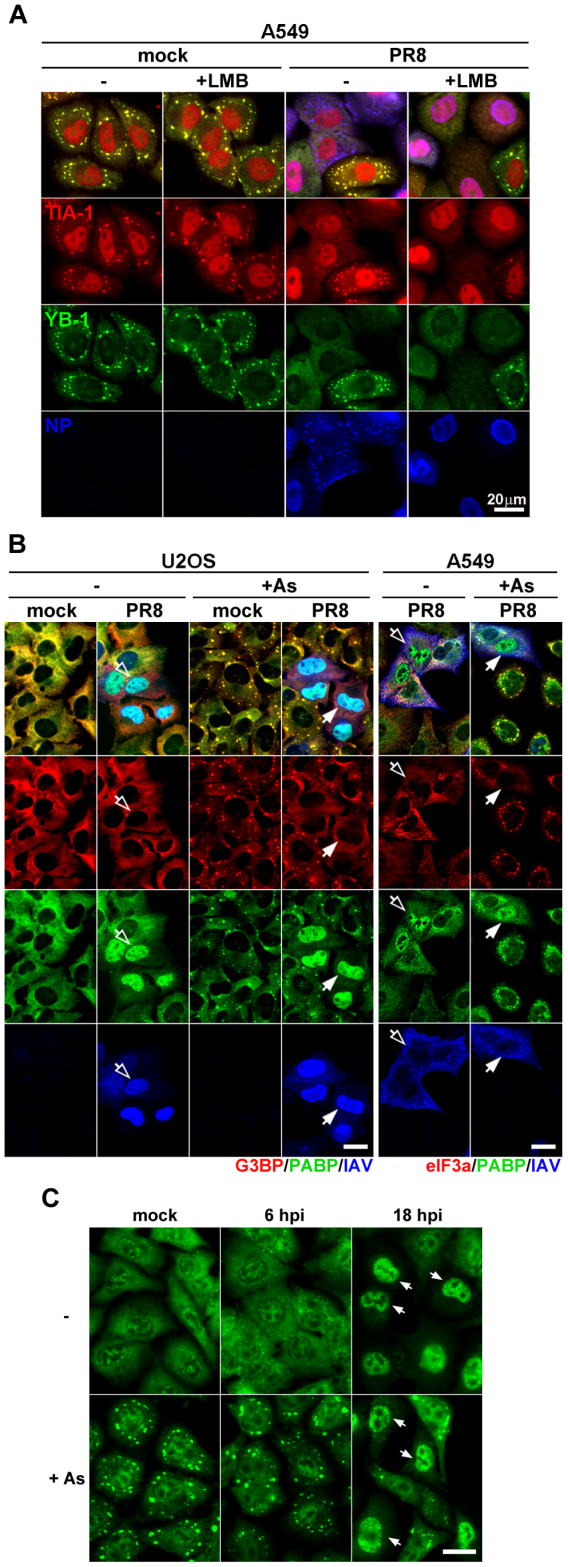
vRNP export-independent SG inhibition by influenza virus coincides with depletion of cytoplasmic poly(A) RNA and nuclear relocalization of PABP1. (A and B) Immunofluorescent analysis of mock and PR8 virus-infected cells at 18 hpi, treated with sodium arsenite where indicated. (A) At 6 hpi some A549 cells received 10 ng/ml leptomycin B (+LMB) to block nuclear export of viral ribonucleoproteins up to the time of arsenite treatment and subsequent fixation at 18 hpi. SG formation was analysed by staining for TIA-1 (red) and YB-1 (green) markers and viral ribonucleoproteins were detected with antibody to NP (blue). (B) SG formation and sub-cellular distribution of cellular G3BP, eIF3A (red), PABP1 (PABP, green), and influenza virus proteins (IAV, blue) were analysed in U2OS and A549 cells. Note that while in U2OS cells most of the IAV antigens remain nuclear at 18 hpi, virus material is exported from the nucleus by this time point in A549 cells (open arrows). In both cell lines, however, virus causes redistribution of PABP1 to the nucleus and the SG formation is blocked (filled arrows). (C) Sub-cellular distribution of poly(A) RNA in mock and PR8 virus-infected A549 cells was analysed at indicated times post-infection by fluorescence in situ hybridization. At 18 hpi, many PR8-infected cells have decreased cytoplasmic and increased nuclear poly(A) signal. Upon arsenite treatment (+As) these cells fail to form cytoplasmic poly(A) RNA-containing stress granules (arrows). Scale bars = 20 µm.

At later times post-infection, IAV vRNPs are exported from the nucleus to the cytoplasm and transit to the cell surface for assembly and egress. We observe inhibition of SG formation late in infection, coincident with the cytoplasmic export of vRNPs. To test whether cytoplasmic accumulation of vRNPs is necessary for SG inhibition, we treated mock- and IAV-infected cells with leptomycin B (LMB), an inhibitor of CRM1-dependent nuclear export. Consistent with previous reports [13], LMB treatment prevented cytoplasmic accumulation of vRNPs (Fig. 2A). Importantly, in our system LMB treatment did not affect SG inhibition in virus-infected cells, and did not alter SG formation in mock-infected cells. Thus, cytoplasmic localization of vRNPs is dispensable for SG inhibition.

Because SGs are comprised of stalled poly(A) mRNAs and certain translation initiation factors, we determined their intracellular location at late times post-infection. We saw a remarkable relocalization of PABP1 to the nucleus of infected cells (Fig. 2B). PABP1 is nucleocytoplasmic shuttling protein that binds newly polyadenylated mRNAs in the nucleus [14]. Thus, the nuclear accumulation of PABP1 strongly suggested that the normal processing and transport of nascent mRNAs is impaired in infected cells, and/or cytoplasmic mRNA pools are severely depleted. Using fluorescence *in situ* hybridization (FISH), we analyzed the nucleocytoplasmic localization of poly(A) mRNA at early and late times post-infection. Subcellular distribution of poly(A) RNA was comparable in mock- and IAV-infected cells at early times post-infection (Fig. 2C and S2). By contrast, at later stages post-infection, we observed striking loss of poly(A) RNA signal from the cytoplasm, and a noticeable increase in the nuclear poly(A) signal (Fig. S2). Importantly, upon arsenite treatment of mock- and IAV-infected cells at early times post-infection, bright cytoplasmic poly(A) foci were observed, consistent with the accretion of mRNAs into SGs. By contrast, no cytoplasmic foci were observed in cells that displayed nuclear accumulation of poly(A) RNA. Taken together, these data suggest that IAV SG inhibition coincides with bulk depletion of cytoplasmic poly(A) mRNA and the nuclear accumulation of PABP1.

### Influenza A virus inhibits SG formation downstream of eIF-2α kinase activation

**Figure 3:**
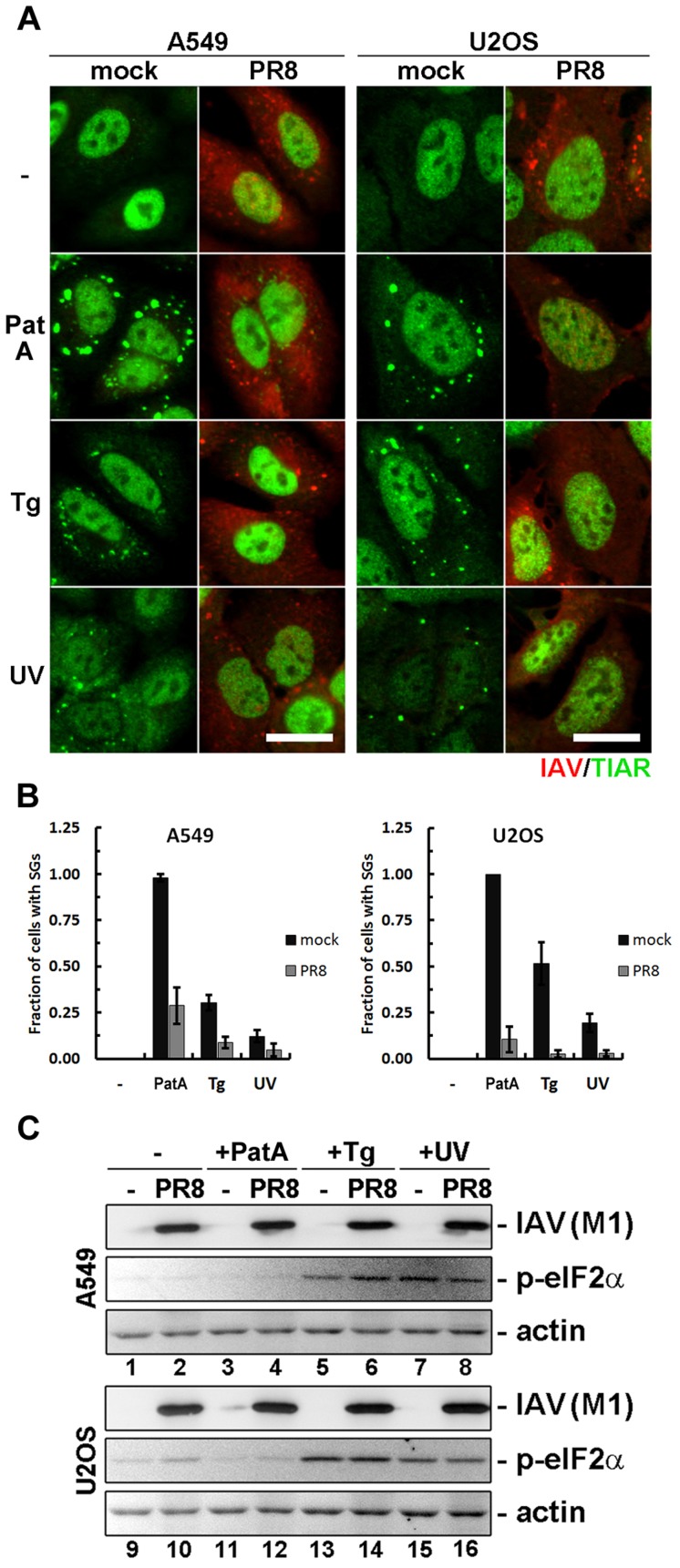
Influenza A virus blocks SG formation independent of inducing stimuli and downstream of eIF2α phosphorylation. SG formation and eIF2α phosphorylation was analysed in mock and PR8 virus infected A549 and U2OS cells at 18 hpi by immunofluorescent staining and western blot. Cells were either left untreated (-), treated for 1 hour with pateamine A (PatA), treated for 1 hour with thapsigargin (Tg), or exposed to UV light and incubated for 2 hours (UV) prior to analysis. (A) Immunofluorescent staining with antibodies to influenza A virus (IAV, red) and TIAR (red). Scale bars = 20 µm. (B) SG formation was quantified from the experiment presented in (A). (C) Western blot analysis of the whole cell lysates of A549 and U2OS cells treated as in (A).

In eukaryotes, eIF2α integrates signals from four stress-activated kinases, and we have established that IAV inhibits SG formation in response to either HRI- or PKR-mediated eIF2α phosphorylation. To determine whether the virus acts downstream of eIF2α phosphorylation, we assessed SG formation triggered by thapsigargin and UV light, which activate the two remaining eIF2α kinases, PERK and GCN2, respectively. As a control, we also tested pateamine A (PatA), which has been shown to induce SGs by translation inhibition but without eIF2α phosphorylation [15] (Fig. 3A–C). In mock-infected cells, these treatments induced varying degrees of SG formation. Nevertheless, consistent with our sodium arsenite data, IAV inhibited SG formation in response to all three treatments without affecting eIF2α phosphorylation (Fig. 3A–C). Most notably, IAV inhibited SG formation in response to PatA treatment, which did not induce eIF2α phosphorylation. Together, these findings establish that IAV can block SG formation in a manner independent of eIF2α phosphorylation.

### Influenza A virus PA and NP ORFs inhibit SG formation

**Figure 4:**
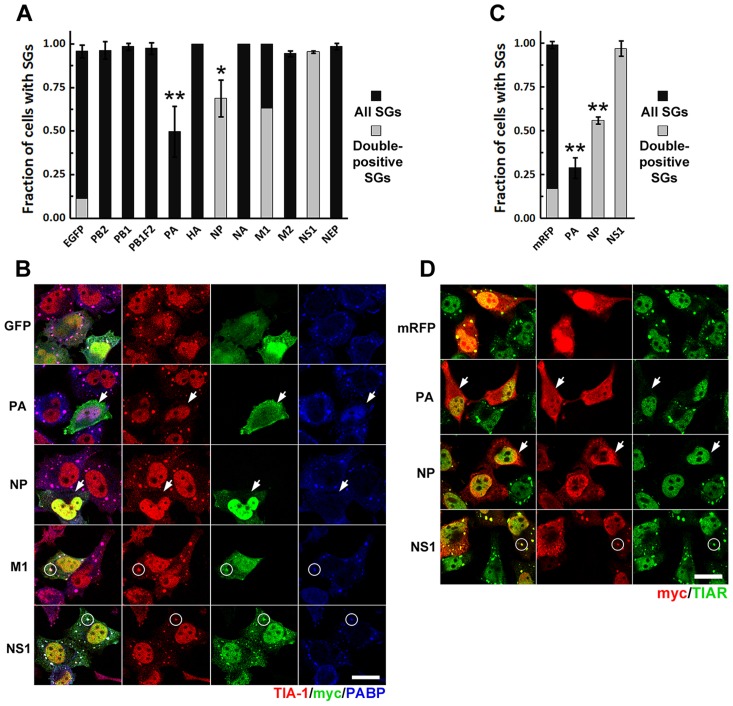
Ectopic expression of influenza A virus PA and NP ORFs inhibits SG formation induced by arsenite and pateamine A. HeLa-TetOff cells were transfected with the expression vectors for the indicated c-terminally myc-tagged IAV ORFs or the EGFP or mRFP expression vectors and treated with sodium arsenite or PatA 24 hours post-transfection. 50 min post-treatment SG formation was analyzed by immunofluorescent staining for TIA-1 or TIAR and SG co-localization of viral ORF-encoded proteins by staining for myc epitope. (A). Arsenite-induced SG formation (dark grey bars) and SG recruitment (double-positive SGs, light grey bar overlays) was quantified from 3 independent experiments. (B). Representative immunofluorescence images demonstrating arsenite-induced SG inhibition by PA-myc and NP-myc (arrows), SG co-localization of M1-myc and NS1-myc proteins (circles), and nuclear PABP1 relocalization in PA-myc expressing cells. (C). PatA-induced SG formation (dark grey bars) and SG recruitment (double-positive SGs, light grey bar overlays) in cells expressing the indicated IAV ORFs and the control mRFP protein was quantified from 2 independent experiments. (D). Representative immunofluorescence images demonstrating SG inhibition by PA-myc and NP-myc (arrows) in response to PatA treatment, and SG co-localization of NS1-myc protein (circles).

To identify additional IAV gene products that could function in this NS1-independent mechanism of SG inhibition, we analyzed arsenite-induced SG formation in cells ectopically expressing myc epitope-tagged constructs for each of the known IAV ORFs (Fig. 4A). Among 11 ORFs analyzed, only PA and NP significantly inhibited arsenite-induced SG formation. PA-myc was predominantly localized to the cytoplasm, consistent with requirement of the PB1 viral subunit for its nuclear import [16]. In cells in which PA failed to suppress SG formation, PA-myc maintained a diffuse cytoplasmic localization and was not recruited to SGs (Fig. 4A). By contrast, NP was found predominantly in the nucleus of transfected cells, with only a fraction of the total signal coming from the cytoplasm. NP-mediated inhibition of SG formation was dependent on NP expression level (Fig. S3), and many cells with low levels of NP formed SGs upon arsenite treatment. In cells in which NP failed to inhibit SG formation, NP was recruited to SGs, consistent with previous reports [17]. Finally, NS1 and the viral protein M1 were similarly recruited to arsenite-induced SGs, but did not interfere with their formation (Fig. 4B). The distinct subcellular distribution and SG-recruitment characteristics of PA and NP suggested distinct mechanisms of SG suppression. Importantly, in cells expressing high levels of PA, SG inhibition coincided with striking relocalization of PABP1 to the nucleus (Fig. 4B), similar to what we observed in IAV-infected cells at late times post-infection (Fig. 2B). Importantly, in both PA-transfected and IAV-infected cells nuclear PABP accumulation invariably coincided with complete inhibition of SG formation in all our experiments. To confirm that both PA and NP interfere with SG formation regardless of eIF2α phosphorylation, we tested PatA-induced SG formation in cells expressing these IAV ORFs (Fig. 4C,D). Both proteins were able to block SG formation in response to PatA. NS1, as expected for this PKR-specific SG inhibitor, did not interfere with arsenite- and PatA-induced SG formation (Fig. 4A–D).

### NP oligomerization is required for efficient SG inhibition

**Figure 5:**
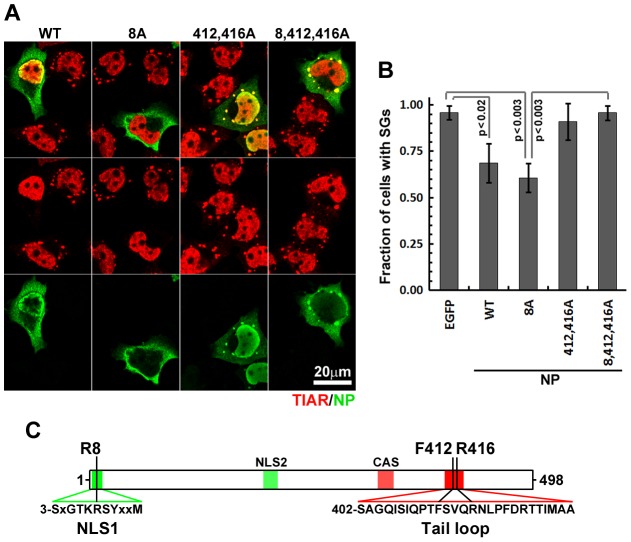
NP oligomerisation is required for SG inhibition. (A and B). HeLa-TetOff cells were transfected with the expression vectors for the indicated wild-type and mutant NP proteins and treated with sodium arsenite 24 hours post-transfection. 50 min post-treatment sub-cellular distribution of NP constructs (green) and SG formation (TIAR, red) was analyzed by immunofluorescent staining (A) and quantified (B). (C). Schematic representation of NP protein showing selected primary sequence features (color shading) and positions of amino acids mutated in this study. Unlike N-terminal nuclear localization signal (NLS1), downstream NLS (NLS2) is believed to be non-functional in the folded protein. The cytoplasmic accumulation sequence (CAS) harbours residues that participate in oligomerisation.

NP associates with IAV genomic and anti-genomic segments and is essential for vRNP synthesis, genome trafficking and virus assembly [18]. Extensive mutational analysis and atomic-resolution structural studies have identified important structural features of NP, including an amino-terminal nuclear-localization signal (NLS) and a carboxy-terminal oligomerization motif [19]–[21], both critical for NP interaction with nascent vRNPs in the nucleus (Fig. 5C). Mutation of arginine 8 to an alanine residue (R8A) prevented nuclear localization of NP, but did not impair its ability to suppress arsenite-induced SG formation, suggesting that NP nuclear accumulation is not required for SG inhibition (Fig. 5A, B). NP oligomerization was experimentally disrupted by substitution of key resides in the tail loop (F412A, R416A) required for the formation of intermolecular salt bridges [19], [20]. Disruption of NP oligomerization completely restored arsenite-induced SG formation in transfected cells (Fig. 5A, B). During infection, NP oligomerization is essential for polymerase activity and for the stability and transport of vRNPs, but our experiments clearly demonstrate that NP-mediated inhibition of SG formation is independent of virion RNAs or polymerase subunits. NP has not been shown to interact with host RNAs, but a number of high-affinity interactions of IAV ribonucleoproteins with host factors have been identified [22], many of which could plausibly be dependent on the ability of NP to oligomerize.

### PA-X mediated depletion of cytoplasmic mRNA coincides with SG inhibition

**Figure 6:**
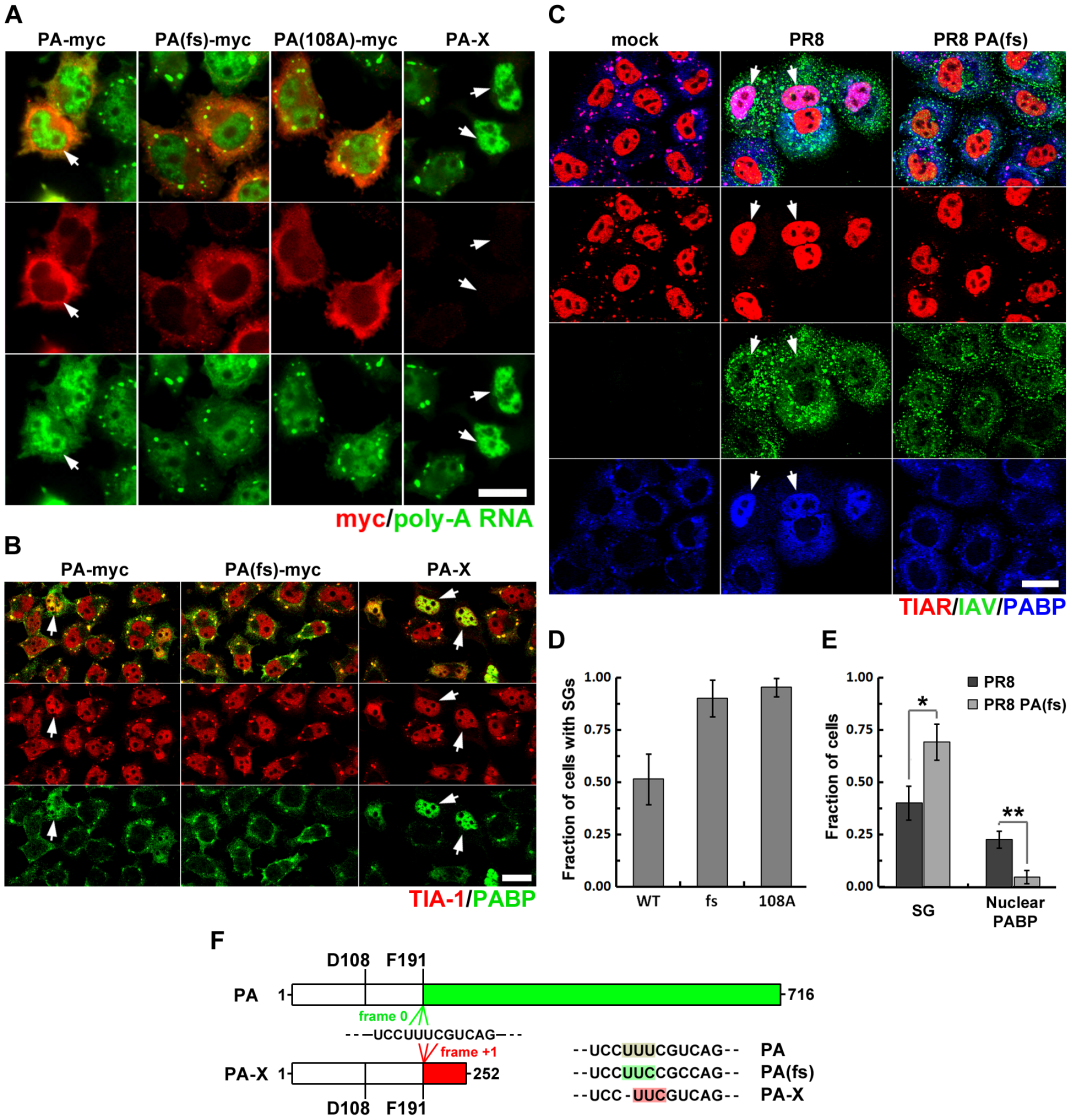
Viral PA-X protein participates in SG inhibition by depleting host cytoplasmic mRNAs. (A, B, D) HeLa-TetOff cells were transfected with the indicated expression vectors and treated with sodium arsenite 24 hours post-transfection. (A) Sub-cellular distribution of poly(A) RNA was analyzed by fluorescence in situ hybridization (green). Expression of the full-length wild type and mutant PA constructs was detected by subsequent immunofluorescent staining for myc epitope (red). (B) SG formation (TIA-1, red) and sub-cellular distribution of PABP1 (PABP, green) was analyzed by immunofluorescent staining. (D) SG formation was quantified from experiments described in (A). (C and E) Immunofluorescent analysis of sodium arsenite-treated mock, wild-type (PR8), and recombinant PA-X deficient (PR8 PA(fs)) virus-infected A549 cells reveals that PA-X expression is required for nuclear PABP1 relocalization and efficient SG inhibition (18 hpi). (C) Representative images showing strong SG inhibition and nuclear PABP1 relocalization in the wild-type, but not the PR8 PA(fs) mutant virus-infected cells treated with sodium arsenite at 18 hpi. (E). Quantification of SG formation and nuclear PABP1 localization from (C). (F). Schematic representation of PA and PA-X proteins synthesized from the same segment 3 transcript and sharing first 191 amino-acids encompassing the endonuclease domain. Continued ORF translation (frame 0, green) produces full-length viral polymerase subunit PA, while +1 frameshifting at F191 codon (frame +1, red) produces smaller PA-X protein. Position of D108 residue that is required for endonuclease activity of PA and PA-X is indicated on the diagram. To the right, frame shift site sequences mutagenized in this study are presented. Note that single nucleotide deletion in PA-X construct completely prevents full-length PA protein expression, as evidenced by the lack of myc-tag detection in the right column of panel (A).

Influenza virus infection results in the rapid decline of host protein synthesis, a process referred to as shutoff. The viral PA protein has been implicated in this host shutoff, but the mechanism remained obscure until the recent discovery of an additional protein, PA-X, produced from the PA gene by ribosomal frameshifting [4] (Fig. 6F). PA-X was shown to inhibit cellular gene expression and modulate viral virulence and the global host response [4], [23]. The PA-myc plasmid shown above to inhibit arsenite-induced SG formation and cause nuclear accumulation of PABP1 is predicted to also encode PA-X (Fig. 4). To determine whether the full-length PA protein, or the alternative PA-X protein product, is responsible for SG inhibition and PABP1 relocalization, we transiently transfected HeLa Tet-Off cells with a carboxy-terminal epitope-tagged PA expression vector, or the same construct with optimized codons for F191 and R192 that would greatly reduce the probability of frameshifting and PA-X production (PA(fs)-myc), or a frameshift mutant constructed to express PA-X exclusively (PA-X, Fig. 6F). Results with these plasmids strongly indicate that the PA-X protein produced as a result of ribosomal frameshifting is responsible for the SG inhibition and PABP1 relocalization observed in PA-myc transfected cells. In cells transfected with the PA-X construct, we observed the strongest nuclear accumulation of PABP1, which was completely concordant with SG inhibition. By contrast, in PA(fs)-myc transfected cells, we could not detect any relocalization of PABP1, and arsenite-induced SG formation was unperturbed. As expected, the PA-myc construct displayed an intermediate phenotype, consistent with a lower expected level of PA-X production (Fig. 6B). To directly confirm that PA-X ORF is expressed in cells showing nuclear PABP relocalisation, we created an additional expression construct with a C-terminally tagged PA-X ORF. Due to high toxicity of PA-X, only cells expressing low levels of this fusion protein remained viable at 24 h post-transfection. Nevertheless, these levels were sufficient to cause nuclear relocalisation of PABP1 and complete blockade of SG formation in response to arsenite treatment (Fig. S4). In the study by Jagger et al. (2012), PA-X expression was linked to host gene expression shutoff, which required an endonuclease function of the N-terminal domain shared by the full-length PA and PA-X proteins (Fig. 6F). It was proposed that this endonuclease activity of PA-X is responsible for depletion of host cytoplasmic mRNA pools. To determine whether PA-X endonuclease activity is responsible for depletion of cytoplasmic PABP1 (Fig. 2B) and poly(A) mRNA (Fig. 2C) late in IAV infection, we disrupted the endonuclease activity of PA and PA-X by a single amino acid substitution at D108 (PA(108A)-myc), and analyzed SG formation and subcellular distribution of poly(A) RNA using FISH (Fig. 6A, D). Similar to PA(fs)-myc transfected cells, cells transfected with PA(108A)-myc displayed normal cytoplasmic levels of poly(A) RNA and formed SGs in response to sodium arsenite.

To examine the contribution of PA-X to the SG-inhibition phenotype at late times post-infection, we created a recombinant IAV with a codon-optimized PA ORF, termed PR8 PA(fs), expected to have an attenuated host-shutoff phenotype [4]. At 18 hpi, the PR8 PA(fs) infected cells showed dramatically decreased nuclear PABP1 relocalization and attenuated SG inhibition in response to sodium arsenite (Fig. 6C, E). Notably, a fraction of cells infected with PR8 PA(fs) remained refractory to arsenite-induced SG formation, perhaps due to inhibition of SG formation by NP protein.

### Pateamine A induces SG formation and blocks influenza A virus replication

**Figure 7:**
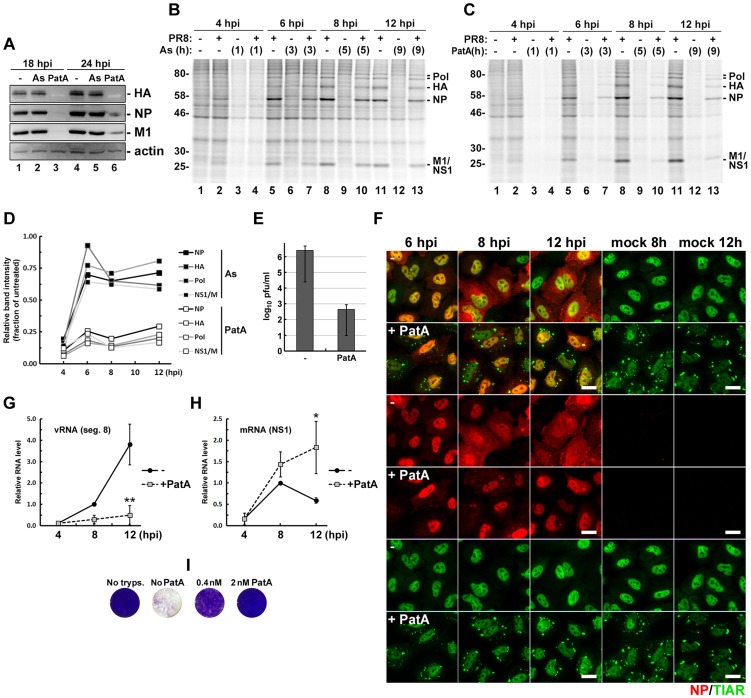
Pateamine A treatment causes sustained arrest of viral protein and genomic RNA synthesis. (A). Effects of 1-hour arsenite and pateamine A treatments on IAV protein accumulation. A549 cells were infected with PR8 virus and treated with either arsenite or pateamine A at 4 hpi. After 1 hour, drug-containing media was removed, cells were washed with PBS and received fresh media. Whole cell lysates were collected at 18 and 24 hpi and analysed by western blot. (B and C) Autoradiograms of the lysates of mock and PR8 virus-infected cells. At 3 hpi cells were either left untreated (-) or treated with sodium arsenite (B) or pateamine A (C) for 1 hour and then pulse-labelled with [^35^S]methionine-[^35^S]cysteine immediately after treatment and at the indicated 2-hour intervals. (D) Recovery of viral protein translation post-arsenite and post-pateamine A treatments was quantified from autoradiograms in (B) and (C). Band intensities from post-treatment lanes were normalized to untreated sample band intensities for each time point. (E to H). SG formation, viral RNA accumulation, and infectious virus production were analysed in mock and PR8 virus-infected A549 cells treated with 10 nM pateamine A at 4 hpi. (E) Virus titers were analysed in culture media collected at 24 hpi by plaque assays. (F) Cells were fixed and immunofluorescently stained for influenza NP (red) and TIAR (green) at indicated times post-infection (hpi). (G) Accumulation of viral genomic RNA segment 8 was quantified by real-time quantitative RT-PCR. (H) Accumulation of viral NS1 mRNA was quantified by real-time quantitative RT-PCR. (I) Pateamine A protects cells from influenza virus-induced cell death. Vero cells were inoculated with PR8 virus at MOI 0.1. After 1 h, inoculums were replaced with infection medium containing 2.5 µg/ml trypsin and the indicated concentrations of pateamine A, or control media lacking trypsin (No tryps.). 2 days post-infection cells were fixed with 4% paraformaldehyde and stained with crystal violet (purple).

We have established that at later stages of its replication cycle IAV efficiently blocks SG formation downstream of eIF2α phosphorylation, while early in infection IAV is particularly sensitive to treatments that induce translation arrest and SG formation (Fig. 1C, F). This early part of the infectious cycle may therefore provide a window of opportunity for pharmacological induction of viral translation arrest and SG formation. Among the treatments that we have tested, we observed strongest induction of SG formation in response to sodium arsenite (Fig. 1) and PatA (Fig. 3). To test the effects of these drugs on viral replication, we pulse-treated IAV-infected cells at 4 hpi, with subsequent removal of drug after 1 h. This 1 h treatment with PatA was sufficient to completely block IAV replication, as evidenced by lack of accumulation of viral proteins at later times post-infection (Fig. 7A). Interestingly, sodium arsenite had minimal effects on IAV protein accumulation. Despite the high toxicity of sodium arsenite, upon its removal cells are reported to rapidly dephosphorylate eIF2α, dissolve SGs, and resume robust translation [24]. By contrast, PatA binds and inhibits the function of eIF4A helicase irreversibly [25]. These properties could explain the differential effect of these SG-inducing agents on viral replication. To test this, we compared translation rates in mock- and PR8-infected cells immediately post-treatment with sodium arsenite or PatA, and at regular intervals post-drug withdrawal. As shown in Fig. 7B, D, soon after removal of sodium arsenite, production of IAV proteins was largely restored. By contrast, translation rates remained low long after PatA was removed (Fig. 7C, D). This sustained inhibition is in agreement with lack of viral protein accumulation up to 14 hours after removal of PatA-containing medium (Fig. 7A), and is marked by the accumulation and persistence of SGs, both in mock- and PR8-infected cells (Fig. 7F). Importantly, persistence of SGs prevented the accumulation of progeny vRNPs in the cytoplasm (Fig. 7F), viral genomic RNA accumulation (Fig. 7G), and infectious virus production (Fig. 7E). By contrast, PatA treatment did not prevent viral mRNA accumulation, and even increased its abundance at later times post-infection (Fig. 7H). This finding is consistent with the model in which viral replication is dependent on *de novo* protein synthesis [26], and earlier observations that treatment with translation inhibitors prevents viral polymerase from initiating genome replication while still allowing viral mRNA synthesis [27].

Given the potency of PatA for IAV inhibition, we tested the effects of low-dose PatA treatment on multi-round IAV infection and observed an inhibitory effect at 2 and 0.4 nM (Fig. 7I). It is notable that we observed no adverse effects of PatA on cell viability at these low doses, consistent with published observations in other model systems [28].

## Discussion

**Figure 8:**
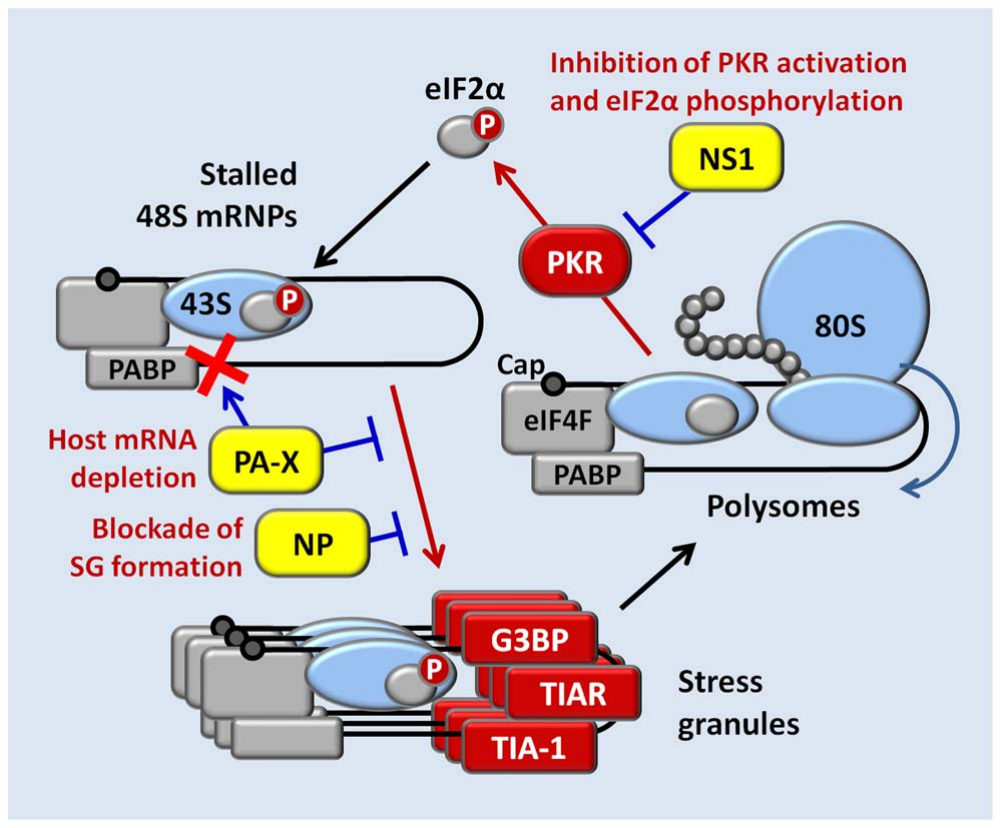
A model for concerted action of NS1, NP, and PA-X proteins in the inhibition of translation arrest and SG formation in influenza A virus-infected cells.

Despite being generated by a viral RNA-dependent RNA polymerase complex, IAV mRNAs bear structural similarity to the products of host RNA polymerase II, with 5′ caps and 3′ poly(A) modifications that ensure efficient translation by host machinery [1]. These features render IAV mRNAs susceptible to negative regulation by eIF2α kinases that have evolved to sense a variety of environmental stresses, including viral infection [5]. Triggering of eIF2α phosphorylation during IAV infection would therefore be expected to arrest translation of a large fraction of viral mRNAs as well as host mRNAs, and entrap them in SGs. Remarkably, we observed that SGs do not form at any point during the IAV replication cycle and efficient translation of viral gene products is maintained in the later stages of infection, when a variety of eIF2α-kinase activating signals might be anticipated. Here we have identified three IAV proteins capable of inhibiting SG formation: NS1, NP and PA-X (Fig. 8). NS1 binds to viral dsRNA and prevents PKR activation and consequent eIF2α phosphorylation. High levels of oligomeric NP block SG formation in an eIF2α-independent manner, through a yet to be identified mechanism. By contrast, inhibition of SG formation by PA-X, the alternative product of PA mRNA that is made as a result of translational frameshifting, is dependent on its endoribonuclease activity, and coincides with depletion of a large fraction of cytoplasmic poly(A) RNA. During PA mRNA translation, less than 2% of the ribosomes frame-shift at the Phe-191, downstream of the endoribonuclease domain that is shared between PA and PA-X proteins [4]. This low rate of frame-shift ensures slow accumulation of PA-X, the potent shutoff factor that acts at later times post-infection. Indeed, mutations that inhibit frame-shifting and PA-X production by IAV disrupt the viral inhibition of host gene expression [4]. Our finding that PA-X inhibits SG formation led us to explore PA-X host shutoff function, and our findings support the recently proposed model; we find that PA-X causes striking depletion of poly(A) RNA from the cytoplasm of cells transfected with a PA-X expression vector, or in IAV-infected cells late in infection, but not in cells infected with a mutant virus with defects in PA-X protein production. Notably, in addition to the depletion of cytoplasmic poly(A) RNA and nuclear relocalization of PABP1, we also observe a remarkable accumulation of poly(A) RNA in the nuclei of cells expressing PA-X and in virus-infected cells at later times post-infection. While this phenomenon has not been demonstrated previously for IAV infections, a similar accumulation of poly(A) RNA and PABP1 has been reported in other viral infections, and in each case the described perturbations of PABP1 has been linked to host shutoff [9]–[11]. For example, host shutoff in cells infected by Kaposi's sarcoma-associated herpesvirus (KSHV) is enforced by the shut-off exonuclease protein SOX, which accumulates during infection and degrades host cytoplasmic mRNAs [29]. SOX expression leads to strong nuclear accumulation of PABP1 and hyper-adenylated mRNAs. It was demonstrated that relocalization of PABP1 to the nucleus results in stimulation of poly(A) polymerase and synthesis of extended poly(A) tails [30]. Given the general inhibition of host mRNA synthesis by IAV, it is plausible that an increase in nuclear poly(A) RNA signal is due to hyperadenylation rather than a blockade of nascent mRNA export, similar to the hyperadenylation observed in cells ectopically expressing KSHV SOX protein or a mutant PABP1 that is restricted to the nucleus [31]. PABP1 localizes to SGs, but is not required for SG formation [6], [32]. Therefore, it remains to be determined whether PA-X directly affects nucleocytoplasmic shuttling of PABP1, or whether PABP1 nuclear accumulation simply results from bulk depletion of cytoplasmic poly(A) RNA. In either case, we have demonstrated here that the endonuclease activity of PA-X is important for SG inhibition, forging the first links between host shutoff and SG dynamics.

Host shutoff is an important feature of influenza virus infection. In human-adapted strains of IAV, host shutoff is mediated at least in part by the NS1 proteins that are able to bind and inactivate the cellular cleavage and polyadenylation specificity factor 30 (CPSF30). This inactivation prevents polyadenylation and nuclear export of host pre-mRNAs [2]. The host shutoff mechanisms employed by IAV strains with NS1 proteins that lack CPSF30-binding activity remain poorly characterized, but the recent discovery of the PA-X protein significantly advances our understanding of host shutoff by these viruses. Curiously, our study reveals that PA-X expression causes nuclear accumulation of poly(A) RNA, which would require polyadenylation of at least some RNA species in the nucleus. At present, we cannot predict the cumulative effects on sub-cellular poly(A) RNA distribution of CPSF30 inactivation by NS1 and nuclear PABP1 relocalization by PA-X, because the NS1 of PR8 strain employed in this study does not bind CPSF30. Interestingly, the PA segment of WSN/33 strain of IAV, which is competent for CPSF inactivation, was less effective at inhibiting host protein synthesis than the PA segment from A/California/04/2009 strain [23]. This difference suggests that the host-shut-off function of PA-X, similar to that of NS1, may vary significantly between strains of IAV.

NP is a highly conserved RNA-binding protein that plays essential roles in IAV transcription, genome replication, and genome packaging into nascent virions. NP binds to RNA with high affinity (Kd approx. 20 nM), but there is little evidence for sequence specificity, and no links have yet been made to the control of mRNA translation [33], [34]. Our data demonstrate that ectopic expression of NP alone, in the absence of viral RNA or other viral proteins, is sufficient to inhibit arsenite-induced SG formation. Structural studies have shown that NP forms oligomers in physiological ionic conditions [19]. The formation of these oligomers depends on intermolecular salt bridges, and we demonstrate that experimental disruption of these salt bridges by substitution of key residues in the tail loop (F412A, R416A) disabled the SG-inhibition function of NP. We are unable to assess the impact of these mutations on NP function during viral infection because they prevent IAV transcription and genome replication [35]. Proteomic studies have identified a number of host proteins bound by viral ribonucleoproteins [22], several of which play roles in mRNA processing, stability and translation. In addition, a number of proteins recruited to SGs and involved in their formation have been shown to bind NP [36], [37], [38]. NP interacts with nuclear factor 90 (NF90) that participates in virus-induced PKR activation and SG formation [38]. Another host antiviral factor that is recruited to SGs and interacts with both NP and NS1 is the RNA-associated protein 55 (RAP55) [36]. When unable to block SG formation (e.g. at early times post-infection) NS1 and NP can be sequestered in SGs by RAP55 where their normal functions are likely to be inhibited. Thus, it is possible that IAV NS1 and NP can be both the antagonists and the targets of host antiviral SG responses. Recently, RNA-dependent interaction of IAV NP with Fragile X mental retardation protein (FMRP) was demonstrated and FMRP was shown to stimulate viral ribonucleoprotein assembly [37]. Unlike the antiviral factors NF90 or RAP55, FMRP contributes to efficient IAV replication, and may be sequestered through recruitment to SGs. This would further impair viral replication and contribute to the antiviral effect of SGs. In our ongoing studies, we plan to employ WT and mutant NP constructs to screen for interactions with these and other host factors, and determine their relative impact on SG dynamics.

We have established that early in infection, before significant accumulation of NP and PA-X proteins, IAV is particularly sensitive to treatments that induce translation arrest and SG formation. This may provide a window of opportunity for pharmacological induction of viral translation arrest and SG formation. Among the treatments tested, we observed strongest induction of SG formation in response to arsenite and to PatA. Until now, PatA and related structural analogs have been evaluated for their antitumor properties, and found to represent promising and selective anti-tumor agents [39], [40]. Like tumor cells, IAV-infected cells require high levels of translation to support their metabolic needs. Moreover, at the concentrations used in our studies, PatA inhibits the translation of only a fraction of cellular RNAs, specifically ones important for tumor cell growth and metastasis [39]. Ongoing *in vitro* studies will address the mechanism of action of pateamine A and other translation inhibitor drugs in IAV restriction.

In summary, we report that translation of IAV proteins continues uninterrupted until late in infection, long after the viral polymerase complex has been re-tasked to viral genome replication. We report the remarkable discovery of three functionally distinct IAV-encoded inhibitors of SG formation, which together strongly influence the translational landscape of infected cells. The existence of three distinct mechanisms of IAV-mediated SG inhibition reveals the magnitude of the threat (to the virus) of stress-induced translation arrest during viral replication, and hints that other viruses, large and small, may also encode multiple SG-regulatory proteins. Investigation of the mechanisms of action of these viral SG inhibitors may reveal important clues about how these viruses enforce host shutoff and ensure preferential production of viral gene products.

## Materials and Methods

### Reagents and cells

Unless specifically indicated, all chemical reagents were purchased from Sigma. Pateamine A was a kind gift from Dr. Jerry Pelletier (McGill University, Montreal, QC, Canada). HeLa-TetOff cells were purchased from Clontech. A549, U2OS, Vero, and HeLa-TetOff cells were maintained in Dulbecco's modified Eagle's medium (DMEM; HiClone) supplemented with 10% fetal bovine serum (FBS, Life Technologies) and 100 U/ml penicillin+100 µg/ml streptomycin+20 µM L-glutamine (Pen/Strep/Gln; Wisent) at 37°C in 5% CO_2_ atmosphere.

### Viruses, infections and treatments

The A/PuertoRico/8/34/(H1N1) virus (PR8) and the recombinant mutant viruses PR8 NS1 38A,41A and PR8 NS1 96,97A are described in [8]. To generate NS1 deletion mutant viruses PR8 NS1 N80 and PR8 NS1 N15, the plasmid encoding segment 8 of PR8 [41] was subjected to Phusion PCR mutagenesis (Finnzymes/Thermo Scientific) to introduce termination codons at amino acid positions 81 and 16, respectively, in the NS1 open reading frame. Resulting constructs were combined with 7 wild-type PR8 plasmids for rescue of the mutant viruses as described in [41], producing PR8 mutants expressing either just the dsRNA binding domain of NS1 (PR8 NS1 N80) or completely lacking functional NS1 and expressing only 15 N-terminal amino acids shared between NS1 and NEP upstream of the unaltered transcript splice site (PR8 NS1 N15). Recombinant virus PR8 PA(fs) was generated using the general approach described above. In the segment 3 rescue plasmid, sequence TTTCGT encoding Phe-191 and Arg-192 of the PA ORF was substituted with the optimized codon sequence TTCCGC to generate the recombinant virus with strongly attenuated +1 ribosomal frame shifting during PA mRNA translation. Mutagenesis primer sequences are available upon request. Virus stocks used for experiments were produced and titrated by plaque assays as described [8]. Genomic RNA segments 3 and 8 of each virus stock were verified by sequencing.

For virus infection experiments, unless specified otherwise, infections were done at multiplicity of infection (MOI) of 0.5. After inoculation, cells received infection medium containing 0.5% bovine serum albumin (BSA) in DMEM and incubated at 37°C in 5% CO_2_ atmosphere. When indicated, mock and virus-infected cells were treated with 0.75 mM sodium arsenite, 10 nM pateamine A (except when other concentrations are given), or 1 µM Thapsigargin. For UV treatment, cells were washed briefly with PBS, exposed to 10,000 µJ/cm^2^ of 254 nm light in HL-2000 Hybrilinker chamber (UVP) and promptly returned to 37°C incubator.

### Plasmids and primers

Rescue plasmids encoding 8 segments of PR8 virus (pHW191-PB2 to pHW198-NS) were kindly provided by Dr. Richard Webby (St. Jude Children's Research Hospital, Memphis, TN, USA). pCR3.1-Myc and pCR3.1-NS1-myc expression vectors are described in [8]. Remaining expression vectors for c-terminally tagged PR8 ORF library (with the exception of M2) were constructed by inserting PCR-amplified coding sequences from the rescue vectors between KpnI and Mlu sites of pCR3.1-Myc vector (flanking restriction sites were introduced by PCR). M2 expression vector was generated by removal of the intervening intron sequence from pHW197-M plasmid using Phusion PCR mutagenesis protocol. Untagged NP expression vector was generated by inserting the PCR-amplified coding sequences from the pHW195-NP plasmid into pCR3.1 vector (Invitrogen/Life Technologies) between EcoRI and XhoI sites. Subsequent substitutions in the NP ORF were done by PCR mutagenesis to produce pCR3.1-NP(8A), pCR3.1-NP(412,416A) and pCR3.1-NP(8,412,416A) vectors. pCR3.1-PA(fs)-myc, pCR3.1-PA(108A)-myc and pCR3.1-PA-X were generated by PCR mutagenesis of the pCR3.1-PA-myc vector from the PR8 ORF library. Cloning primer sequences are available upon request.

### Transient transfection

HeLa- TetOff cells were seeded at a density of 100,000 cells/well of a 12-well culture plate and transfected the next day with 0.5 µg total DNA/well using FuGene HD reagent (Promega) according to manufacturer's protocol. At 24 h post-transfection, cells were treated as described and analysed by immunofluorescent staining.

### Immunostaining

Cells grown on glass coverslips were fixed and immunostained according to the protocol in [6] using mouse monoclonal antibodies to IAV nucleoprotein (AAH5; Abcam), G3BP (clone 23, BD Transduction Labs), and PABP1 (sc-32318, Santa Cruz Biotechnology); goat polyclonal antibody to TIA-1 (sc-1751, Santa Cruz Biotechnology), influenza virus (ab20841, Abcam), rabbit antibody to TIAR (clone D32D3, Cell Signaling), YB-1 (ab12148, Abcam) and myc-tag (9B11; Cell Signaling) at manufacturer-recommended dilutions. AlexaFluor-conjugated secondary antibodies (Molecular Probes) were used at 1∶1000 dilution. Images were captured using Zeiss Axioplan II microscope or Zeiss LSM 510 laser scanning microscope. Quantification of stress granules was done in at least 3 random fields of view with greater than 200 cells analysed on each slide. Cells were considered stress granule-positive if two or more stress granule marker foci were present in the cytoplasm. For western blot analysis, whole cell lysates were resolved on denaturing 10% polyacrylamide gels and analyzed using primary antibodies described above and the antibodies to phospho-Ser-51- eIF2α (rabbit, D9G8, Cell Signaling), eIF4A (goat, sc-14211, Santa Cruz Biotechnology), and actin (rabbit, 4968; Cell Signaling).

### Isotopic labelling

As described in [8].

### Fluorescence in situ hybridisation

Cells grown on glass coverslips were washed briefly with PBS, fixed with 4% paraformaldehyde in PBS for 15 min at room temperature, and permeabilized using 0.1% Triton X-100 in PBS for 10 min. Coverslips were equilibrated in wash buffer (2× SSC, 20% formamide, 0.02% BSA) for 15 min and then incubated overnight with 100 µl of hybridization mix (wash buffer with 10% dextran sulphate, 0.4 mg/ml *S. cerevisiae* tRNA, and 100 nM Fluorescein-conjugated oligo-dT-40 probe) at 42°C. Then, coverslips were washed in wash buffer for 15 min at 42°C, for 15 min at room temperature, and finally with PBS for 15 min prior to either mounting on slides or blocking for subsequent immunostaining.

### Real time quantitative PCR

Total RNA was isolated using the RNeasy mini-prep kit (Qiagen) according to the manufacturer's instructions, and the cDNA synthesis was performed using the Super Script III Reverse Transcriptase Kit (Life Technologies). Quantitative PCR analysis was performed using MX6000P unit (Agilent Technologies) and GoTaq PCR master mix (Promega). For analysis of NS1 mRNA reverse transcription was performed using oligo-dT(20) primer. For reverse transcription of segment 8 vRNA the following primer was used: 5′-CAA ACA CTG TGT CAA GCT TTC AG. Same primer set was used for the mRNA and vRNA-derived cDNA amplification: NS1-Left 5′-CTG TGT CAA GCT TTC AGG TAG A and NS1-Right 5′- GGT ACA GAG GCC ATG GTC AT. Relative initial template quantities were determined using the standard curve method. For each biological replicate, reactions were done in duplicate and mean cycle threshold (Ct) values were used.

### Statistical analyses

All error bars represent standard deviations calculated from values obtained in at least 3 independent biological replicates. Analysis of variance (ANOVA) single-factor algorithm was applied to the select datasets using Microsoft Excel data analysis module (Microsoft) to calculate p values. Where indicated, asterisks denote p values lower than 0.05 (*) or 0.005 (**).

## Supporting Information

Figure S1 **Influenza A virus blocks SG formation in response to sodium arsenite downstream of eIF2α phosphorylation.** Stress granule formation and eIF2α phosphorylation were analysed in mock and PR8 virus-infected U2OS cells treated with sodium arsenite. (A to L) Immunofluorescent staining for SG marker G3BP (red) and influenza virus antigens (PR8, green) at indicated times post-infection (hpi). Uninfected and some virus-infected cells that formed SGs in response to arsenite are highlighted with arrows. (M) SG inhibition was quantified from the experiment presented in panels (A–L). (N) Western blot analysis of mock and PR8 virus-infected U2OS cells treated with sodium arsenite at indicated times post-infection. (O) Western blot analysis of cellular translation factors expression in PR8 virus-infected cells at indicated times post-infection.
(TIF)

Figure S2 **Inhibition of SG formation in IAV-infected cells correlates with the redistribution of poly(A) RNA to the nucleus and the decrease in host mRNA levels.** (A and B). Cytoplasmic and nuclear poly(A) RNA fluorescence in situ hybridization signal in untreated and arsenite-treated mock and PR8-infected A549 cells was measured using Image J software (imagej.nih.gov). Outlines for the cytoplasm and the nucleus of each individual cell were selected manually and the mean signal intensities for the green channel were quantified. At least 3 images of randomly-selected fields of view were used to quantify signals from 15 cells in each category. Because only some PR8-infected cells formed SGs after arsenite treatment at 18 hpi, cells that formed SGs at 18 hpi and those that remained SG-free were grouped in two separate categories. (A). No significant changes in either cytoplasmic (left panel) or nuclear (middle panel) signal intensities were observed between mock-infected and PR8-infected cells at 6 hpi. Similarly, the ratios between nuclear and cytoplasmic signals determined for each cell (right panel) did not change significantly between these categories. By contrast, significant reduction of cytoplasmic signal and corresponding increase in nuclear signal was observed in infected cells at 18 hpi compared to mock-infected cells. Importantly, at 18 hpi, in cells that did not form SGs upon arsenite treatment, cytoplasmic signals were significantly lower, and the nuclear signals were significantly higher, than in cells that formed SGs. (B) Untreated (top panel) and arsenite-treated (lower panel) PR8-infected cells at 18 hpi, analysed by fluorescence in situ hybridization for subcellular distribution of poly(A) RNA. Representative outlines of nuclear (Nuc.) and cytoplasmic (Cyt.) areas used to measure mean signal intensities presented in panel (A) are shown for some cells. Filled arrows indicate cells that had measurable redistribution of poly(A) RNA signal to the nucleus (nuclear to cytoplasmic ratio above 2.5) and did not form SGs upon arsenite treatment. Cells that formed arsenite-induced SGs are indicated with open arrows. Scale bars = 20 µm. (C). Levels of host actin and tubulin mRNAs, as well as viral NS segment vRNA, were compared by RT-qPCR in PR8-infected cells between 6 and 18 hpi. Values for host transcripts were plotted relative to levels in mock-infected cells, whereas NS vRNA levels were plotted relative to 6 hpi. All values were normalized to total RNA levels. Primers for amplification of host actin and tubulin cDNAs were ACTB-Left: 5′-CAT CCG CAA AGA CCT GTA CG; ACTB-Right 5′-CCT GCT TGC TGA TCC ACA TC; TUBB-Left: 5′-TCT ACC TCC CTC ACT CAG CT; and TUBB-Right: 5′-CCA GAG TCA GGG GTG TTC AT. Primers for NS vRNA reverse transcription and qPCR amplification are described in Materials and methods. (D). RNA harvested at the indicated time points was visualized using denaturating agarose gel electrophoresis and ethidium bromide staining. 28S and 18S rRNA band intensities were used for normalization of RT-qPCR data presented in (C).
(TIF)

Figure S3 **Inhibition of arsenite-induced SG formation by NP correlates with its expression level.** (A). A representative image of HeLa-TetOff cells transfected with influenza NP expression vector and treated with sodium arsenite 24 h post-transfection. SG formation was visualized by staining with rabbit anti-eIF3A antibody (D51F4, Cell Signaling, red), and NP expression levels were revealed by staining with mouse anti-NP antibody (AAH5, Abcam, green). Cells that express high levels of NP (filled arrows, numbered 1 to 4) failed to form SGs. Cells with intermediate NP expression levels (open arrows, numbered 5 to 7), low levels of NP (open arrowheads, numbered 8 and 9), and the non-expressing cell (number 10) formed SGs in response to arsenite. Scale bars = 20 µm. (B). NP immunofluorescence signal was measured and plotted for each of the numbered cells on panel (A). Measurement procedure was done using Image J software as described in the legend for figure S2 panels A and B, except the mean signal intensity was determined for the entire cell (nucleus+cytoplasm).
(TIF)

Figure S4 **Low levels of PA-X expression are sufficient for SG inhibition and nuclear relocalization of PABP.** HeLa-TetOff cells transiently transfected with the indicated expression constructs were treated with sodium arsenite at 24 hpi, and immunostained with anti-TIA1 antibody to visualize SGs (TIA-1, red) and anti-PABP1 antibody to determine its subcellular distribution (PABP, blue). Expression of the myc-tagged constructs was visualized using anti-myc antibody. Scale bars = 20 µm.
(TIF)
