# Supplementary figures and images for: Influenza A Virus Host Shutoff Disables Antiviral Stress-Induced Translation Arrest
Source: PLoS Pathog. 2014 Jul 10;10(7):e1004217. doi: 10.1371/journal.ppat.1004217 (PMC4092144; doi:10.1371/journal.ppat.1004217)

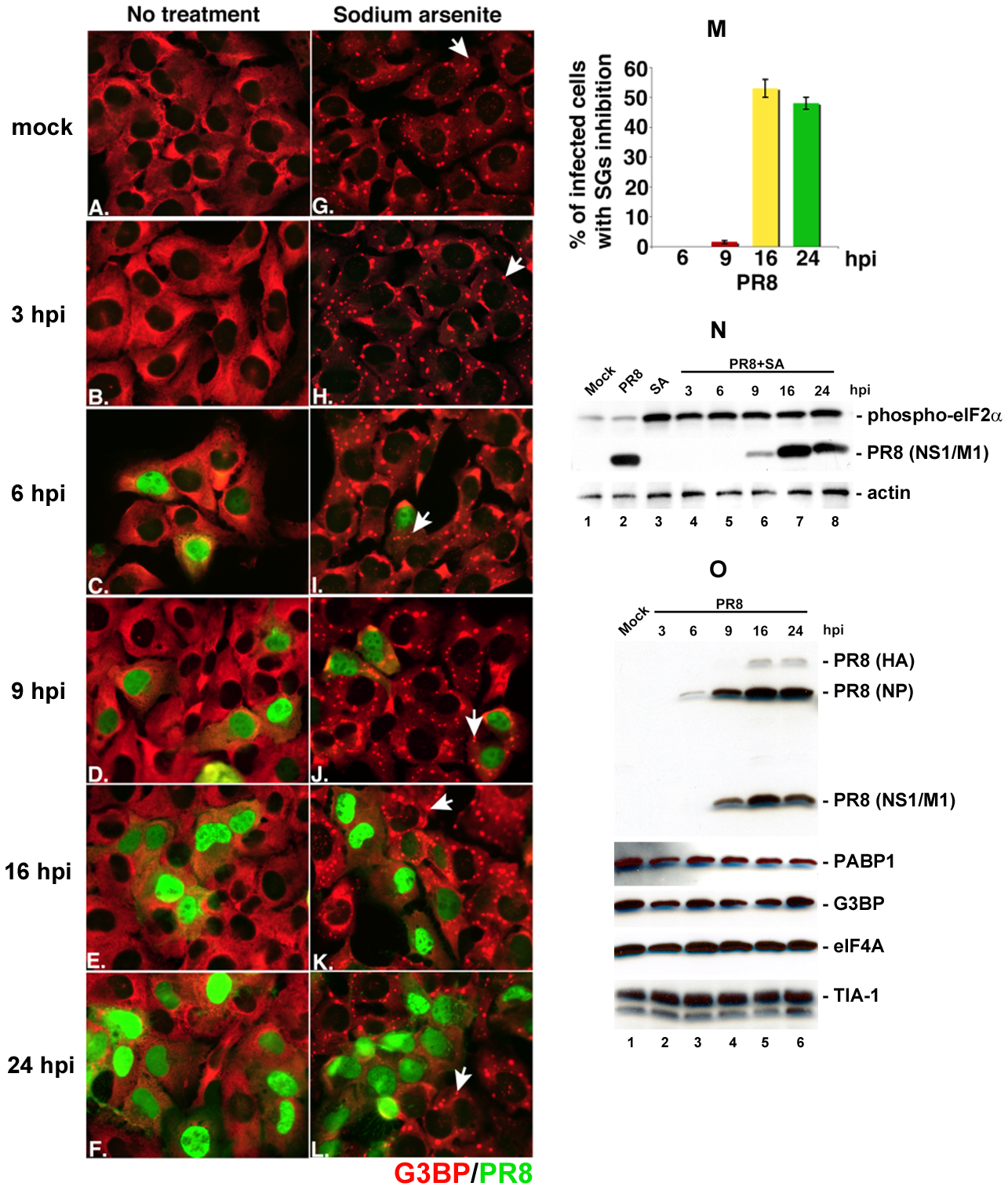

Supplement: Figure S1 — Influenza A virus blocks SG formation in response to sodium arsenite downstream of eIF2α phosphorylation. Stress granule formation and eIF2α phosphorylation were analysed in mock and PR8 virus-infected U2OS cells treated with sodium arsenite. (A to L) Immunofluorescent staining for SG marker G3BP (red) and influenza virus antigens (PR8, green) at indicated times post-infection (hpi). Uninfected and some virus-infected cells that formed SGs in response to arsenite are highlighted with arrows. (M) SG inhibition was quantified from the experiment presented in panels (A–L). (N) Western blot analysis of mock and PR8 virus-infected U2OS cells treated with sodium arsenite at indicated times post-infection. (O) Western blot analysis of cellular translation factors expression in PR8 virus-infected cells at indicated times post-infection. (TIF) [file ppat.1004217.s001.tif]

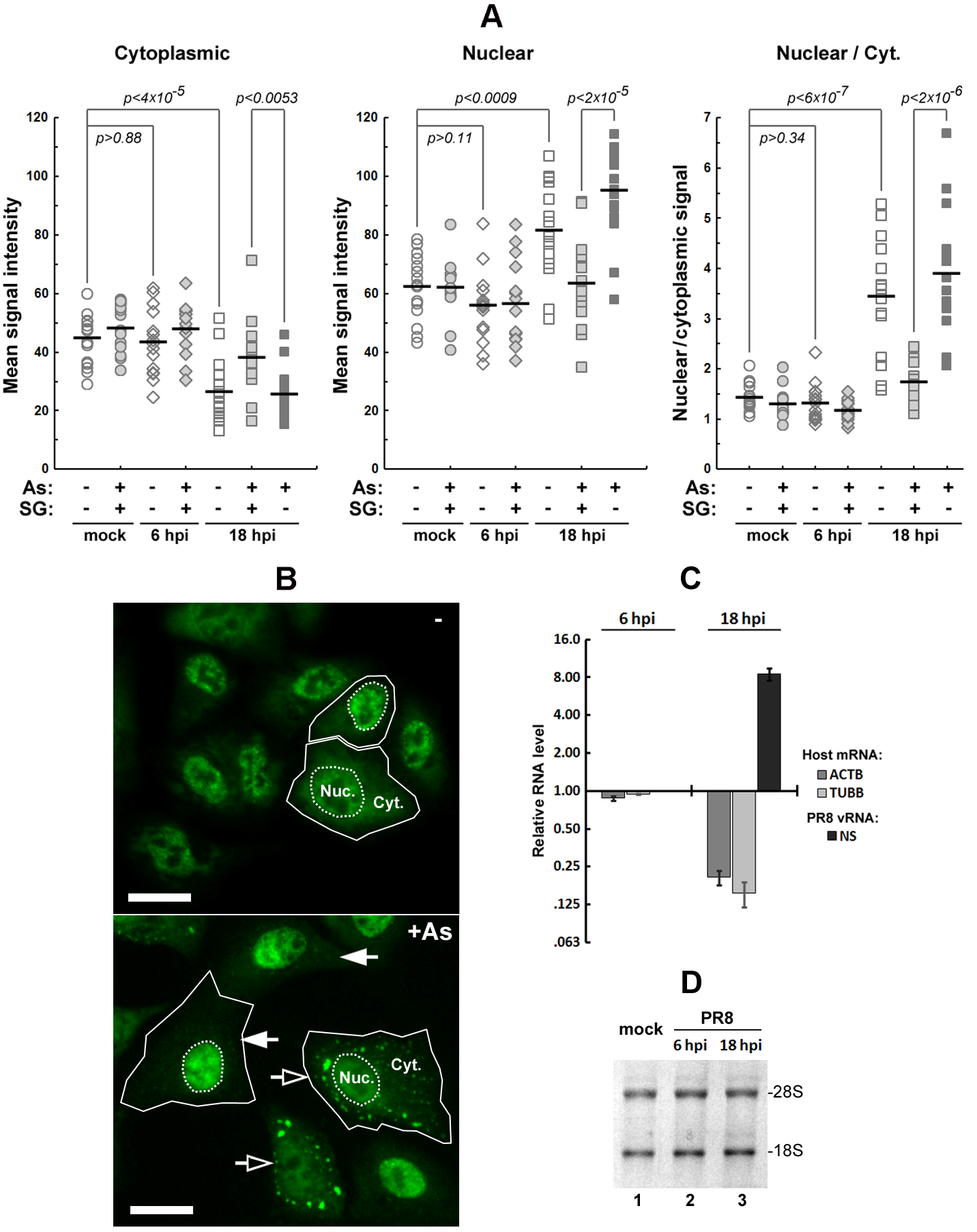

Supplement: Figure S2 — Inhibition of SG formation in IAV-infected cells correlates with the redistribution of poly(A) RNA to the nucleus and the decrease in host mRNA levels. (A and B). Cytoplasmic and nuclear poly(A) RNA fluorescence in situ hybridization signal in untreated and arsenite-treated mock and PR8-infected A549 cells was measured using Image J software (imagej.nih.gov). Outlines for the cytoplasm and the nucleus of each individual cell were selected manually and the mean signal intensities for the green channel were quantified. At least 3 images of randomly-selected fields of view were used to quantify signals from 15 cells in each category. Because only some PR8-infected cells formed SGs after arsenite treatment at 18 hpi, cells that formed SGs at 18 hpi and those that remained SG-free were grouped in two separate categories. (A). No significant changes in either cytoplasmic (left panel) or nuclear (middle panel) signal intensities were observed between mock-infected and PR8-infected cells at 6 hpi. Similarly, the ratios between nuclear and cytoplasmic signals determined for each cell (right panel) did not change significantly between these categories. By contrast, significant reduction of cytoplasmic signal and corresponding increase in nuclear signal was observed in infected cells at 18 hpi compared to mock-infected cells. Importantly, at 18 hpi, in cells that did not form SGs upon arsenite treatment, cytoplasmic signals were significantly lower, and the nuclear signals were significantly higher, than in cells that formed SGs. (B) Untreated (top panel) and arsenite-treated (lower panel) PR8-infected cells at 18 hpi, analysed by fluorescence in situ hybridization for subcellular distribution of poly(A) RNA. Representative outlines of nuclear (Nuc.) and cytoplasmic (Cyt.) areas used to measure mean signal intensities presented in panel (A) are shown for some cells. Filled arrows indicate cells that had measurable redistribution of poly(A) RNA signal to the nucleus (nuclear to [file ppat.1004217.s002.tif]

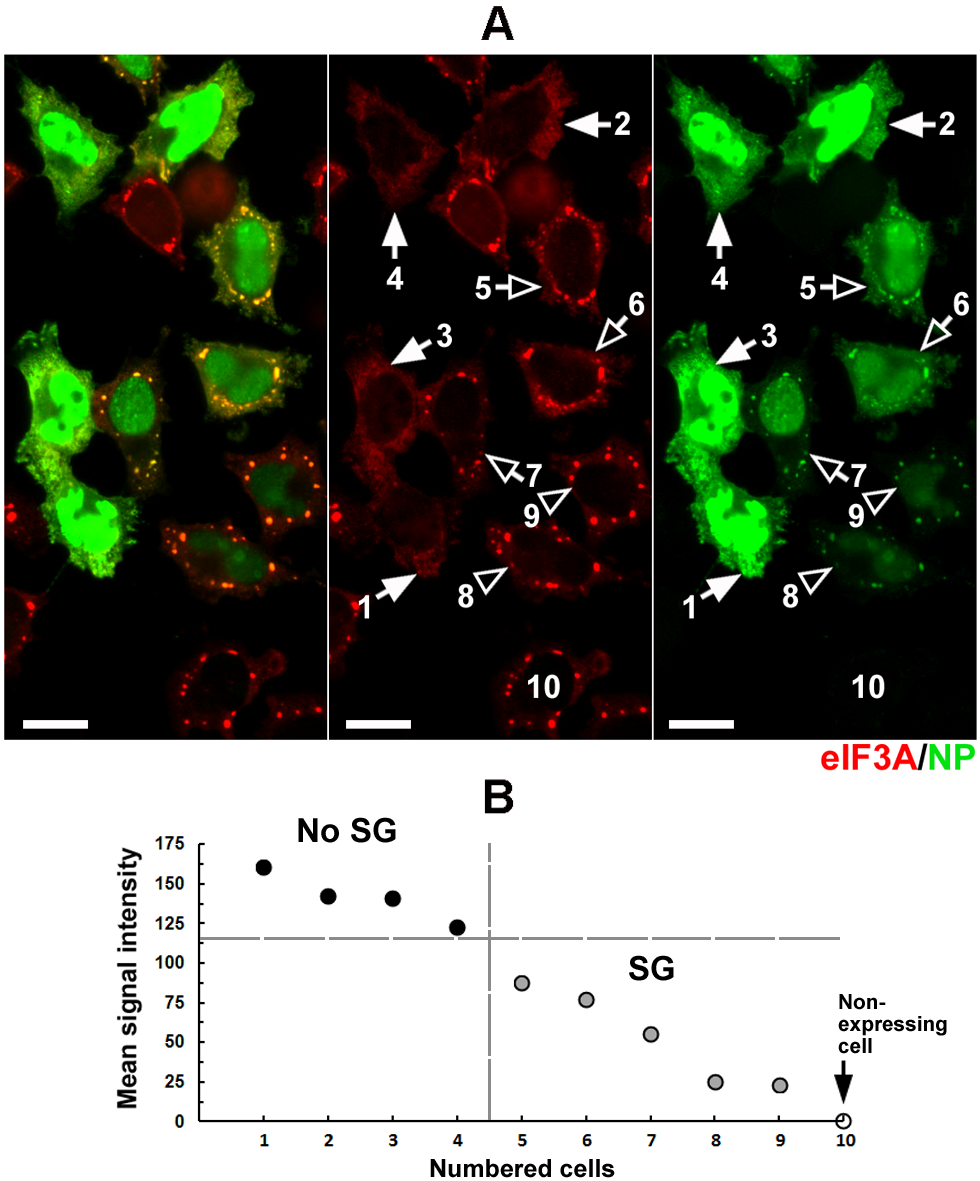

Supplement: Figure S3 — Inhibition of arsenite-induced SG formation by NP correlates with its expression level. (A). A representative image of HeLa-TetOff cells transfected with influenza NP expression vector and treated with sodium arsenite 24 h post-transfection. SG formation was visualized by staining with rabbit anti-eIF3A antibody (D51F4, Cell Signaling, red), and NP expression levels were revealed by staining with mouse anti-NP antibody (AAH5, Abcam, green). Cells that express high levels of NP (filled arrows, numbered 1 to 4) failed to form SGs. Cells with intermediate NP expression levels (open arrows, numbered 5 to 7), low levels of NP (open arrowheads, numbered 8 and 9), and the non-expressing cell (number 10) formed SGs in response to arsenite. Scale bars = 20 µm. (B). NP immunofluorescence signal was measured and plotted for each of the numbered cells on panel (A). Measurement procedure was done using Image J software as described in the legend for figure S2 panels A and B, except the mean signal intensity was determined for the entire cell (nucleus+cytoplasm). (TIF) [file ppat.1004217.s003.tif]

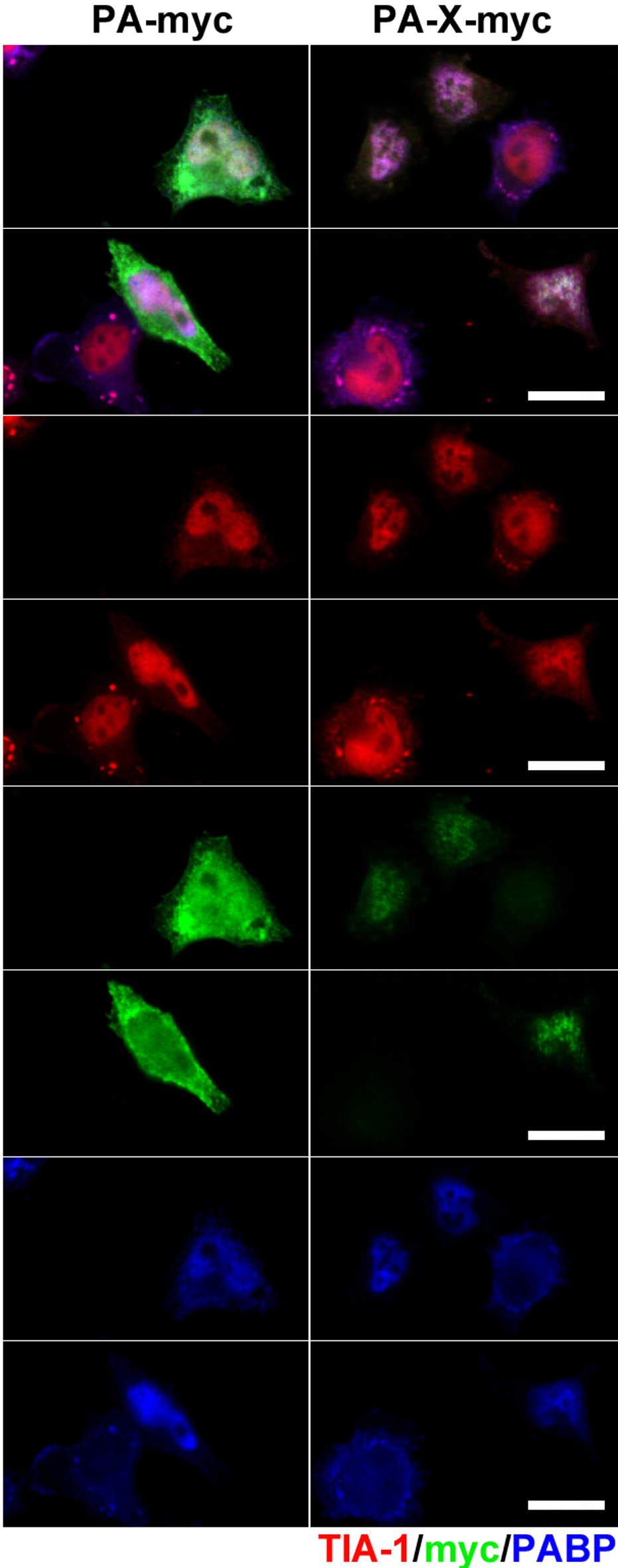

Supplement: Figure S4 — Low levels of PA-X expression are sufficient for SG inhibition and nuclear relocalization of PABP. HeLa-TetOff cells transiently transfected with the indicated expression constructs were treated with sodium arsenite at 24 hpi, and immunostained with anti-TIA1 antibody to visualize SGs (TIA-1, red) and anti-PABP1 antibody to determine its subcellular distribution (PABP, blue). Expression of the myc-tagged constructs was visualized using anti-myc antibody. Scale bars = 20 µm. (TIF) [file ppat.1004217.s004.tif]
